# The IκB Kinase Complex Is Required for Plexin-B-Mediated Activation of RhoA

**DOI:** 10.1371/journal.pone.0105661

**Published:** 2014-08-19

**Authors:** Matthias Zielonka, Ramesh K. Krishnan, Jakub M. Swiercz, Stefan Offermanns

**Affiliations:** 1 Max-Planck-Institute for Heart and Lung Research, Department of Pharmacology, Bad Nauheim, Germany; 2 Institute of Pharmacology, University of Heidelberg, Heidelberg, Germany; 3 Medical Faculty, University of Frankfurt, Frankfurt am Main, Germany; Karolinska Institutet, Sweden

## Abstract

Plexins are widely expressed transmembrane proteins that mediate the cellular effects of semaphorins. The molecular mechanisms of plexin-mediated signal transduction are still poorly understood. Here we show that signalling via B-family plexins leading to the activation of the small GTPase RhoA requires activation of the IκB kinase (IKK)-complex. In contrast, plexin-B-dependent regulation of R-Ras activity is not affected by IKK activity. This regulation of plexin signalling depends on the kinase activity of the IKK-complex, but is independent of NF-κB activation. We confirm that the IKK-complex is active in tumour cells and osteoblasts, and we demonstrate that plexin-B-dependent tumour cell invasiveness and regulation of osteoblast differentiation require an active IKK-complex. This study identifies a novel, NF-κB-independent function of the IKK-complex and shows that IKK directs plexin-B signalling to the activation of RhoA.

## Introduction

Plexins constitute a group of receptors which are activated by semaphorins [1], [2]. Semaphorins and plexins are widely expressed [3]–[6], and the semaphorin-plexin system plays important roles during development and in the adult organism. This includes functions in organogenesis, the nervous and immune system as well as in tumour progression and metastasis [7]–[10]. Mammalian plexins are divided into four subfamilies: Plexin-A1–4, Plexin-B1-B3, Plexin-C1 and Plexin-D1 [1]. All plexins possess a GTPase-activating protein (GAP) domain which has activity towards R-Ras, M-Ras and Rap [11]–[13]. B-family plexins in addition mediate an activation of the small GTPase RhoA through their stable interaction with the guanine nucleotide exchange factors PDZ-RhoGEF (Rho guanine nucleotide exchange factor 11) and LARG (Rho guanine nucleotide exchange factor 12) [14]–[16]. B-family plexins are directly activated by semaphorins. While Plexin-B1 responds to Semaphorin 4A (Sema4A) and Sema4D, Plexin-B2 binds Sema4A, Sema4C, Sema4D and Sema4G, and Plexin-B3 is activated by Sema4A and Sema5A [1],[17]–[21]. Semaphorin-induced RhoA activation via B-family plexins requires association of plexin with the receptor tyrosine kinase ErbB-2 [22]. Upon binding of Sema4D to Plexin-B1, ErbB-2 is activated, resulting in tyrosine phosphorylation of Plexin-B1 and ErbB-2 [23]. Phosphorylation of plexin tyrosine residues provides docking sites for SH2 domains, resulting in the recruitment of phospholipase C-γ (PLCγ) into the receptor complex, which is required for the subsequent activation of RhoA through PDZ-RhoGEF [24]. ErbB-2 phosphorylation and RhoA activation are required for several downstream cellular effects including the promigratory and prometastatic effects of semaphorins on cancer cells and Sema4D-induced axonal growth cone collapse [22],[24]. In ErbB-2-overexpressing tumours, ErbB-2 signals through Plexin-B1 and RhoA to promote metastasis [25]. In osteoblasts, Plexin-B1-mediated, ErbB-2-dependent RhoA activation mediates inhibition of osteoblast differentiation induced by Sema4D produced by osteoclasts [26].

We hypothesized that Plexin-B1-mediated RhoA activation involves so far unknown protein kinases and tested the effect of siRNA-mediated knockdown of about 700 mammalian kinases on Sema4D-induced, Plexin-B1-mediated RhoA activation. Here we show that the kinase activity of the IKK-complex is required for the activation of ErbB-2 and RhoA signalling mediated through B-family plexins in response to semaphorins, and we provide evidence that activation of IKK signalling promotes plexin-B signalling in cancer cells and osteoblasts, leading to tumour progression and bone loss, respectively.

## Results

### The IKK-complex is involved in Plexin-B1-mediated RhoA-activation

**Figure 1:**
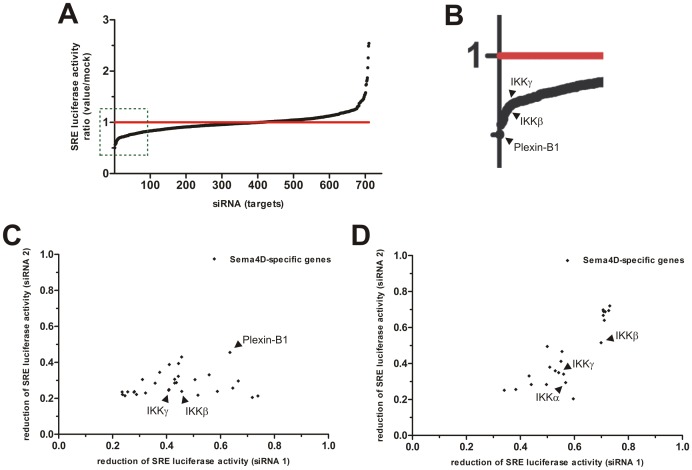
Results of RNAi screen for protein kinases involved in Plexin-B1-mediated RhoA activation. An siRNA library targeting 710 human protein kinases was screened in MCF-7 cells, and the normalized activity of SRE reporter luciferase in response to Sema4D was determined as described in *Materials and Methods*. (**A**) Shown is the ratio of the Sema4D effect on SRE reporter activity in cells transfected with an siRNA pool against a particular human protein kinase and cells transfected with control siRNA. (**B**) Represents magnified boxed area in (A). (**C**) Reduction in Sema4D-induced SRE luciferase activity induced by single siRNAs (siRNA1 and 2) directed against the respective target gene. (**D**) Result of a secondary screen performed with two independent siRNAs per identified target as described in *Materials and Methods*. In addition, siRNAs targeting IKKα were included in the analysis.

To identify novel protein kinases that are functionally relevant in Plexin-B1-mediated downstream signalling, we performed a screen with small interfering RNAs (siRNA) directed against all known human kinases in MCF-7 cells stably expressing firefly luciferase under the control of a mutated serum response element (SRE). In order to determine the effect of siRNA-mediated knockdown on Sema4D-induced, Plexin-B1-mediated activation of RhoA, we used an SRE mutant which lacks the ternary complex factor binding site and responds to signalling downstream of the small GTPase RhoA [27]. In parallel, we determined the effect of siRNAs on SRE activation induced by lysophosphatidic acid (LPA) acting through G-protein-coupled LPA receptors. Since Plexin-B1 and LPA receptor signalling converge on the level of the RhoGEF proteins PDZ-RhoGEF and LARG [15],[16],[28],[29], this approach allowed to sort out hits interfering with RhoGEF activity or any downstream signalling events. In addition, we measured cell viability in each well to detect potentially toxic effects of siRNAs. SiRNAs directed against Plexin-B1 were used as positive controls and strongly reduced Sema4D-induced reporter luciferase activity (Figure 1A and B), thus proving the functionality of the screening procedure. Among 710 kinases screened by siRNA-mediated silencing, the two subunits of the IκB kinase (IKK-) complex, IKKβ and IKKγ, were found among the top candidate genes whose knockdown specifically decreased SRE reporter luciferase activity after stimulation with Sema4D but not with LPA in at least 2 out of 3 experiments (Figure 1A–C). Their involvement in Plexin-B1-mediated signalling could be confirmed by two independent siRNAs per identified target. While the third component of the IKK-complex, IKKα, was not identified in the initial screen, two IKKα-targeting siRNAs tested independently strongly reduced SRE-dependent firefly luciferase expression in response to Plexin-B1 stimulation (Figure 1D), indicating a crucial role of the IKK-complex in Plexin-B1-mediated RhoA activation.

### The kinase activity of the IKK complex is required for plexin-B-mediated ErbB-2 phosphorylation and RhoA activation

**Figure 2:**
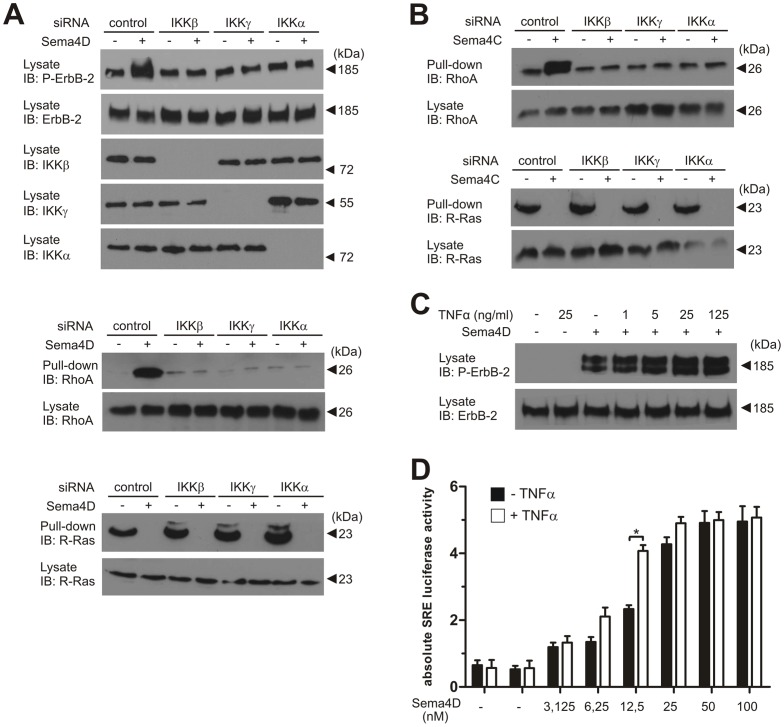
Involvement of the heterotrimeric IKK-complex in cellular signalling via B-plexins. (**A, B**) MCF-7 cells were transfected with control siRNA or siRNAs directed against IKKα, IKKβ or IKKγ. 48 hours after transfection, cells were incubated in the absence (−) or presence (+) of 150 nM Sema4D (A) or Sema4C (B) for 20 minutes, lysed, and the amount of activated RhoA and R-Ras as well as their respective expression levels were determined as described in *Materials and Methods*, or cells were lysed and ErbB-2 phosphorylation was visualised using a specific anti-phospho-ErbB-2 antibody. Equal protein expression levels in cell lysates were confirmed by immunoblotting using an anti-ErbB-2 antibody. (**C**) MCF-7 cells incubated in the absence (−) or presence (+) of 25 nM Sema4D were simultaneously stimulated with increasing concentrations of TNFα for 20 minutes, lysed and ErbB-2 phosphorylation was visualized using a specific anti-phospho-ErbB-2 antibody. Shown are representative examples of at least three experiments. (**D**) MCF-7 reporter cells were treated without (−) or with (+) 25 ng/ml of TNFα. Simultaneously, cells were incubated with increasing concentrations of Sema4D (as indicated) for 8 hours, and SRE luciferase activity was quantified. Shown are the mean values of three indpendent experiments −/+ SD. *, P<0.05.

To further analyze the potential involvement of the IKK-complex in signalling mechanisms mediated by B-family plexins, we examined the effect of siRNA-induced knockdown of IKK-subunits on different B-plexin downstream signalling events. Transfection of siRNAs directed against IKKα, IKKβ or IKKγ blocked Sema4D-induced, Plexin-B1-mediated tyrosine phosphorylation of ErbB-2 and RhoA-activitation in MCF-7 cells. However, the Sema4D-induced increase in GAP activity of Plexin-B1 towards R-Ras was unaffected (Figure 2A), indicating that the depletion of the IKK-complex did not affect the functionality of Plexin-B1 in general. To test whether this role of IKK is restricted to Plexin-B1 or also involves the closely related Plexin-B2, we stimulated MCF-7 cells with Sema4C to activate endogenously expressed Plexin-B2. Analogous to Plexin-B1-mediated effects, depletion of each IKK-subunit almost abolished Sema4C-induced RhoA-activation without affecting R-RasGAP activity of Plexin-B2 (Figure 2B).

We then tested whether activation of the IKK-complex is able to further promote Plexin-B1 signalling. Therefore MCF-7 cells were exposed to increasing concentrations of TNFα. In the presence of a submaximally active concentration of Sema4D, addition of TNFα enhanced Sema4D-induced ErbB-2 phosphorylation (Figure 2C). Also the dose dependence of Sema4D-induced activation of RhoA was slightly shifted to the left in the presence of TNFα (Figure 2D). The effects of TNFα and Sema4D were not additive.

**Figure 3:**
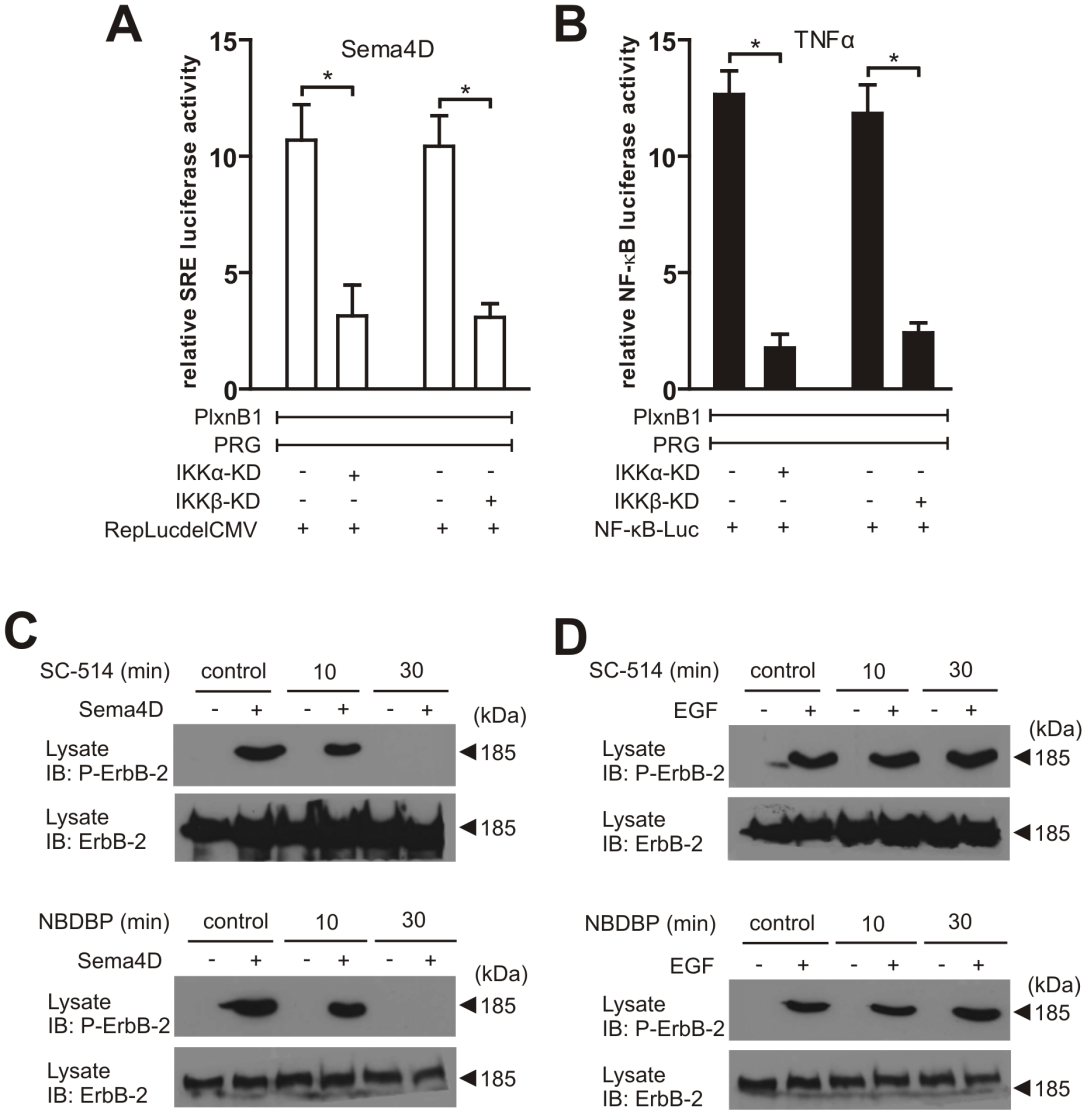
The IKK-kinase activity is required for Plexin-B1-mediated ErbB-2-phosphorylation. (**A, B**) HEK-293 cells were transfected with cDNAs encoding VSV-Plexin-B1 (VSV-PlxnB1), FLAG-PDZ-RhoGEF (PRG) alone or together with kinase-deficient mutants of HA-tagged IKKα (K44M) (IKKα-KD) or HA-tagged IKKβ (K44M) (IKKβ-KD) including SRE.L reporter (RepLucdelCMV) (A) or NF-kB-dependent luciferase reporter plasmid (NF-κB-Luc) (B). 48 hours after transfection, cells were incubated with 25 ng/ml TNFα or 150 nM Sema4D for 8 hours (as indicated), and luciferase activity was determined. Shown are the mean values of three independent experiments −/+ SD. *, P<0.05. (**C, D**) MCF-7 cells were treated with SC-514 (50 µM) or NBDBP (100 µM) for the indicated time periods. After incubation in the absence (−) or presence (+) of 150 nM Sema4D for 20 minutes (C) or 10 ng/ml EGF for 20 minutes (D), cells were lysed, and a specific antibody directed against the phosphorylated version of ErbB-2 was used to visualize ErbB-2 phosphorylation. ErbB-2 levels in lysed samples were controlled using an anti-ErbB-2 antibody. Shown are representative examples of at least three experiments.

Consistent with earlier studies showing that the IKK-complex mainly induced downstream signalling mechanisms by phosphorylation of specific substrates at conserved serine residues through the catalytic subunits IKKβ and IKKα [30], overexpression of kinase-deficient IKKα and IKKβ mutants strongly reduced Sema4D-induced SRE reporter luciferase activity and TNFα-induced NF-κB luciferase activity (Figure 3A and B). Both, SC-514, which interferes with IKKβ-mediated phosphorylation of target proteins by competitive binding to its kinase domain [31], and a cell permeable synthetic peptide, NBDBP, which interferes with the interaction of IKKα/IKKβ and IKKγ, thereby preventing the formation of functional heterotrimeric IKK-complexes [32], blocked Plexin-B1-mediated phosphorylation of ErbB-2 in MCF-7 cells (Figure 3C), but did not affect IKK-independent ErbB-2 phosphorylation in response to stimulation with EGF (Figure 3D). These data indicate that the kinase activity of the IKK-complex is required for ErbB-2 phosphorylation and the subsequent activation of RhoA via B-plexin family members.

### The IKK-complex is not activated in response to Sema4D and regulates B-plexin-mediated signal transduction in an NF-κB-independent manner

**Figure 4:**
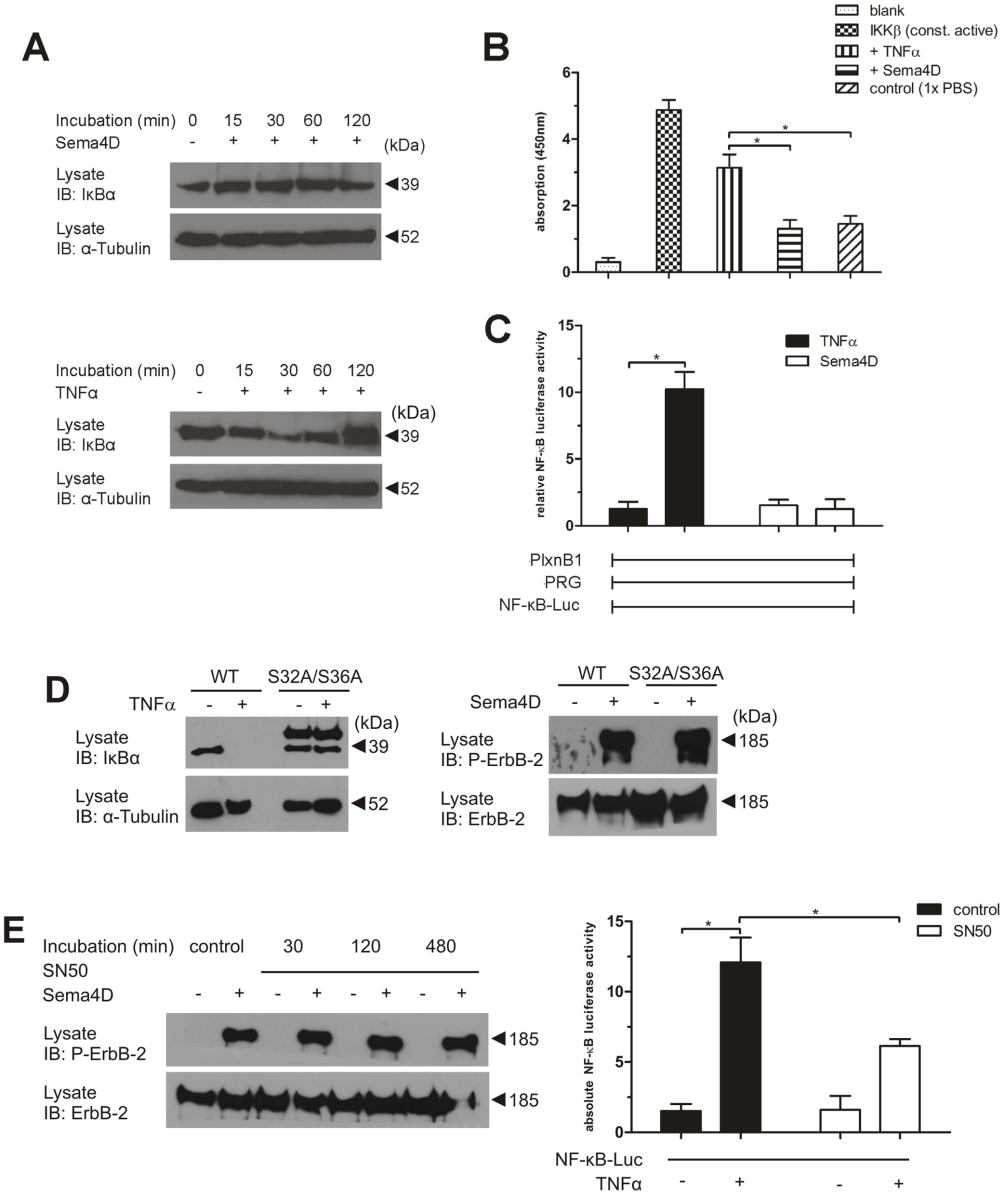
The IKK-complex is not activated by B-plexins and regulates B-plexin-dependent signalling independently of NF-κB. (**A**) After incubation with Sema4D (150 nM) or TNFα (25 ng/ml) for the indicated time periods, MCF-7 cells were lysed, and IκBα degradation was visualized using an anti-IκBα antibody. (**B**) MCF-7 cells were treated with TNFα (25 ng/ml), Sema4D (150 nM) or control buffer (PBS) for 20 minutes and lysed. IKKα/β proteins were precipitated using an anti-IKKα/β antibody. Precipitates were further processed and subjected to an *in vitro* kinase assay as described in *Materials and Methods*. A recombinant active IKKβ isoform served as positive control. Shown are the mean values of absorption measured at a wavelength of 450 nm of three independent experiments −/+ SD. *, P<0.05. (**C**) HEK-293 cells were transfected with cDNAs coding for VSV-tagged Plexin-B1 (VSV-PlxnB1), FLAG-tagged PDZ-RhoGEF (FLAG-PRG) and NF-κB-dependent luciferase reporter plasmid (NF-κB-Luc). 48 hours after transfection, cells were incubated without (−) or with (+) TNFα (25 ng/ml) or Sema4D (150 nM) for 8 hours followed by the photometric quantification of reporter luciferase activity. (**D**) Wild-type MCF-7 cells (WT) and MCF-7 cells transduced with a degradation-resistent dominant-negative IκBα mutant (S32A/S36A) were serum-depleted, incubated in the absence (−) or presence (+) of 25 ng/ml TNFα or 150 nM Sema4D for 20 minutes and lysed. Lysates were probed with anti-IκBα antibody (left panel) to test the expression and functionality of the IκBα mutant or were immunoblotted with an anti-phospho-ErbB-2 antibody to visualize phosphorylated ErbB-2 and with an anti-ErbB-2 antibody to control expression levels (right panel). Protein levels were controlled by immunoblotting with an anti-α-tubulin antibody. (**E**) MCF-7 cells were preincubated with 25 µM of NF-κB inhibitor SN50 for the indicated time periods. Thereafter, cells were treated with control buffer (−) or 150 nM Sema4D (+) for 20 minutes, lysed and ErbB-2 phosphorylation was analyzed as described (left panel). To test the functionality of the NF-κB inhibitor, HEK-293 cells were transfected with a NF-κB dependent luciferase reporter plasmid (NF-κB-Luc) (right panel). After preincubation with 25 µM SN50 for 120 minutes, HEK-293 cells were incubated in the absence (−) or presence (+) of 25 ng/ml TNFα for 8 hours and luciferase acitivity was quantified. Shown are the mean values of three independent experiments −/+ SD. *, P<0.05.

We then tested whether IKKs and other components of the canonical NF-κB pathway are activated by Sema4D-induced Plexin-B1 activation. Whereas TNFα led to a degradation of IκBα, reaching a maximum after 30 minutes, no IκBα degradation was observed in response to Sema4D (Figure 4A). In addition, TNFα but not Sema4D induced an increase in IKKβ kinase activity (Figure 4B) as well as NF-κB activation (Figure 4C).

In the canonical NF-κB pathway, the IKK-complex mediates phosphorylation of IκB-proteins, targeting them for ubiquitination and subsequent proteasomal degradation, thereby liberating NF-κB heterodimers, which translocate into the nucleus and induce the transcription of specific NF-κB dependent genes [33],[34]. To test whether Plexin-B1-mediated signalling depends on the canonical NF-κB pathway downstream of the IKK-complex, we tested the effect of a dominant negative IκBα mutant on Sema4D-induced ErbB-2 phosphorylation.This dominant-negative mutant has serine-to-alanine substitutions at amino acids 32 and 36, respectively, and is resistant to phosphorylation-induced degradation of IκBα, thereby also preventing degradation of endogenous IκBα [35]. While dominant negative IκBα was resistant to TNFα-induced degradation, it had no effect on Sema4D-mediated ErbB-2 phosphorylation (Figure 4D). Furthermore, preincubation of MCF-7 cells with the cell-permeable NF-κB inhibitory peptide SN50 had no effect on Sema4D-induced ErbB-2 phosphorylation (Figure 4E). This indicates that NF-κB activation is not involved in IKK-dependent regulation of Plexin-B1 signalling.

### The IKK-complex is required for the association of Plexin-B1 and ErbB-2

**Figure 5:**
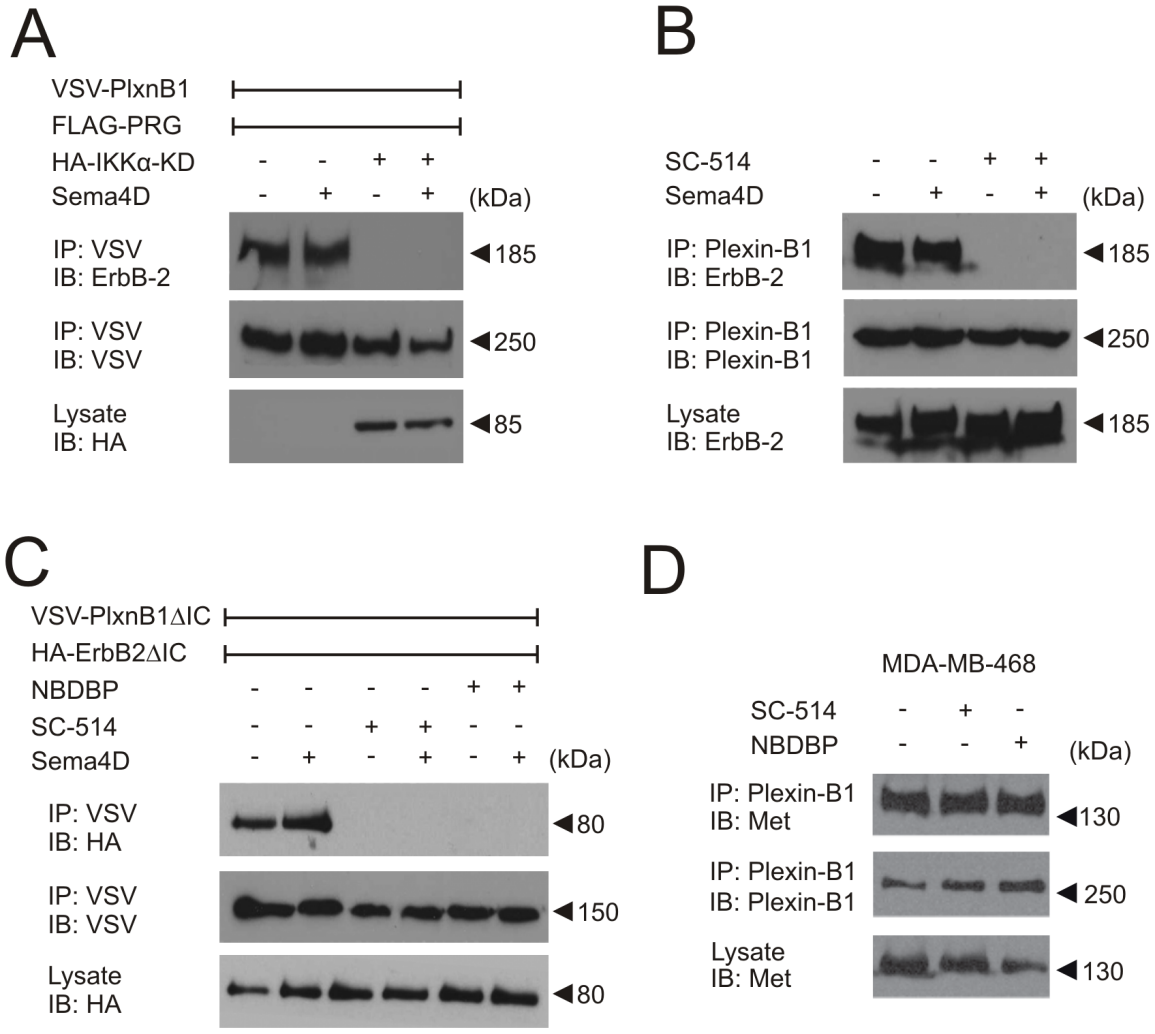
The IKK-complex is required for the interaction of Plexin-B1 with ErbB-2. (**A**) HEK-293 cells were transfected with cDNAs encoding VSV-Plexin-B1 (VSV-PlxnB1), FLAG-PDZ-RhoGEF (FLAG-PRG) alone or together with a HA-tagged kinase-deficient IKKα-mutant (HA-IKKα-KD). After incubation in the absence (−) or presence (+) of 150 nM Sema4D for 20 minutes, VSV-Plexin-B1 was immunoprecipitated (IP) using an anti-VSV antibody and precipitates were immunoblotted (IB) using anti-ErbB-2, anti-VSV or anti-HA antibodies. Shown are the autoluminograms of immunoblots stained with the indicated antibodies. (**B**) MCF-7 cells were incubated with buffer (−) or IKK inhibitor SC-514 (50 µM) for 30 minutes. Thereafter, cells were stimulated without (−) or with 150 nM Sema4D (+) for 20 minutes, lysed, and endogenous Plexin-B1 was immunoprecipitated using an anti-Plexin-B1 antibody. Shown are Western blots of lysed or immunoprecipitated (IP) samples stained with the indicated antibodies (IB). (**C**) 48 hours after transfection with cDNAs encoding truncated versions of VSV-Plexin-B1 (VSV-PlxnB1ΔIC) and HA-ErbB-2 (HA-ErbB-2ΔIC), HEK293 cells were treated without (−) or with IKK inhibitor – SC-514 (50 µM) or NBDBP (100 µM) for 30 minutes. Thereafter, cells were incubated in the absence (−) or presence (+) of 150 nM Sema4D for 20 minutes, lysed and immunoprecipitated (IP) using an anti-VSV antibody. Precipitates were seperated by SDS-PAGE and analyzed by immunoblotting (IB) with anti-VSV- or anti-HA- antibodies. (**D**) MDA-MB-468 cells were incubated without (−) or with SC-514 (50 µM) or NBDBP (100 µM) for 30 minutes, and Plexin-B1 was then immunoprecipitated (IP) from lysed cells. Precipitates were analysed by SDS-PAGE and immunoblotted with antibodies against c-Met or Plexin-B1. Shown are representative examples of at least three experiments.

Given that a blockade of IKK activity affects Plexin-B-mediated ErbB-2 phosphorylation, we tested whether IKK inhibition had an effect on the interaction of Plexin-B1 and ErbB-2. A kinase-deficient IKKα mutant as well as the IKK inhibitor SC-514 blocked coimmunoprecipitation of Plexin-B1 and ErbB-2 in transfected HEK-293 cells as well as in MCF-7 cells, which endogenously express both proteins [23] (Figure 5A and B). Previously, we observed that Plexin-B1- and ErbB-2 mutants lacking the whole intracellular part of the protein can still interact [22]. Interestingly, different IKK-inhibitors also blocked coimmunoprecipitation of truncated ErbB-2 and Plexin-B1 mutants (Figure 5C). Taking into account that the IKK-complex is present in the cytoplasm, this strongly indicates that the IKK-complex inhibits the interaction between Plexin-B1 and ErbB-2 indirectly by phosphorylation of another protein. Consistent with this, we were not able to observe any IKK-dependent phosphorylation of Plexin-B1 or ErbB-2 (data not shown). Since B-family plexins can also interact with other receptor tyrosine kinases, such as c-Met [36], we tested whether the IKK-complex is also required for plexin-Met interaction. We found that in MDA-MB-468 cells, which express endogenous Plexin-B1 and c-Met [23], inhibition of IKK had no effect on the interaction of both receptors (Figure 5D).

**Figure 6:**
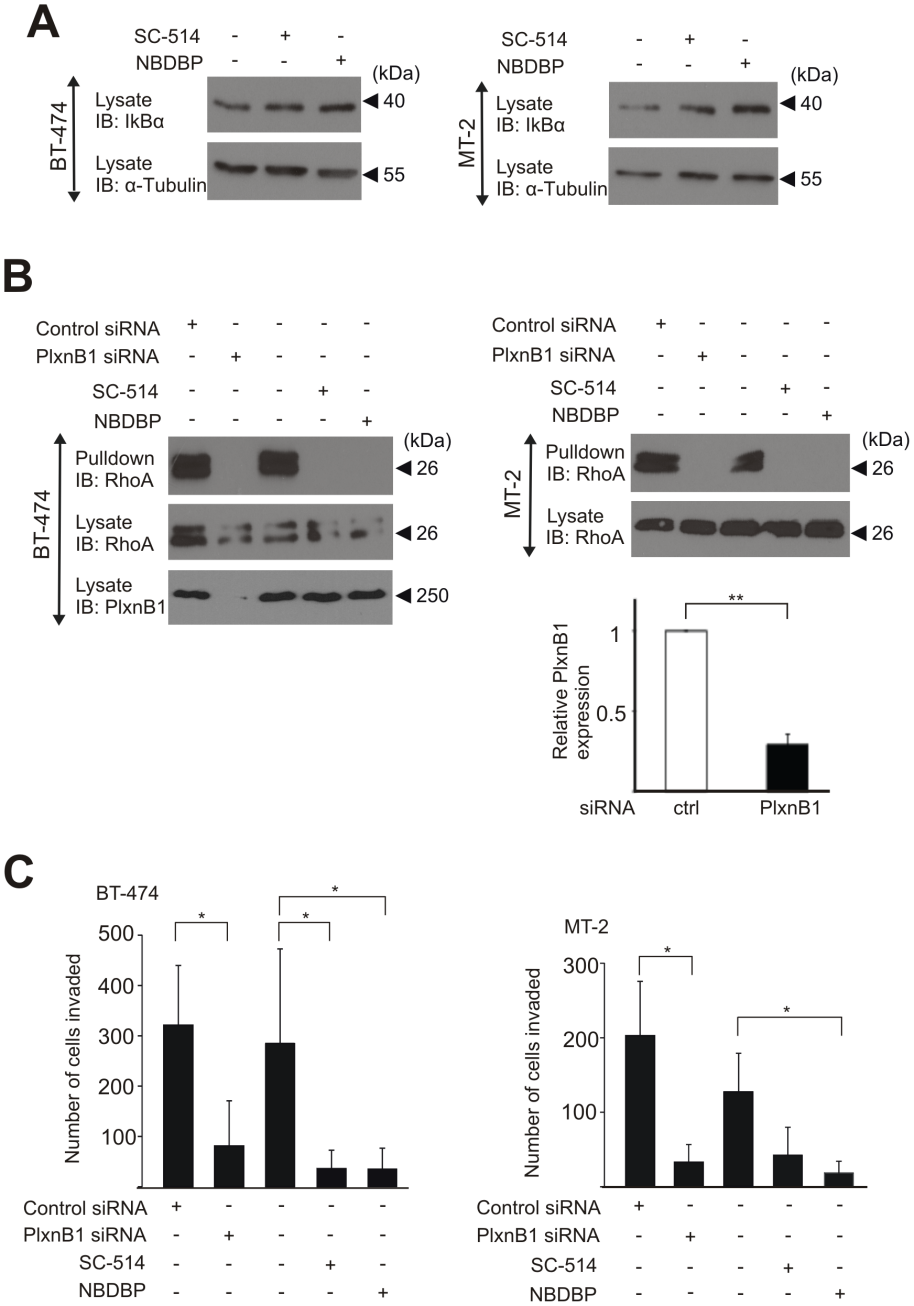
IKK-dependent B-Plexin signalling in ErbB-2-overexpressing tumour cells. (**A**) BT-474 and MT-2 cells were treated with control buffer, 25 µM SC-514 or 13 µM NBDBP for 30 minutes and lysed. Cell lysates were immunoblotted using anti-IκBα and anti-α-tubulin antibodies. (**B**) BT-474 and MT-2 cells were transfected with control or Plexin-B1 siRNA. 48 hours later, cells were starved for 12 hours and treated with IKK-inhibitors as described above. Cells were lysed, and the levels of activated RhoA were determined as described in the *Materials and Methods*. Shown are representative examples of at least three experiments. In a parallel experiment cells were lysed and Plexin-B1 expression in MT-2 cells was tested using RT-PCR. (**C, D**) Non-transfected BT-474 (C) and MT-2 (D) cells or cells transfected with control siRNA or siRNA directed against Plexin-B1 were counted and 1×10^5^ (BT474) or 3×10^4^ (MT-2) cells were plated in transwell invasion inserts. Non-transfected cells were treated without or with IKKβ/γ inhibitors as described. After 24 hours, invaded cells were fixed with 4% PFA, stained with Hoechst 33342 and counted. Data are expressed as mean values −/+ SD from triplicates. *, P<0.05

Consistent with previous reports showing that the IKK-complex is activated constitutively in different cancer cell lines [37]–[43], we could detect elevated IKKβ kinase activity levels and an IKK-dependent IκB degradation under basal conditions in MCF-7, BT-474 and MT-2 cells (Figure 4; Figure 6A). This suggests that constitutively active IKK-complexes in tumour cells prime signalling via B-plexins. To analyze the functional relevance of the observed role of the IKK-complex in signalling through B-family plexins, we studied Plexin-B1-mediated signalling in ErbB-2-expressing cancer cells. The invasive activity of cancer cells overexpressing ErbB-2 is mediated by Plexin-B1 and RhoA [25]. In ErbB-2-overexpressing tumour cells, such as BT-474 and MT-2, RhoA activity was strongly reduced by blockade of the IKK-complex or knockdown of Plexin-B1 (Figure 6B). Consistent with a central role of Plexin-B1 in ErbB-2-induced tumour progression [25], blockade of IKK also reduced tumour cell invasiveness (Figure 6C).

**Figure 7:**
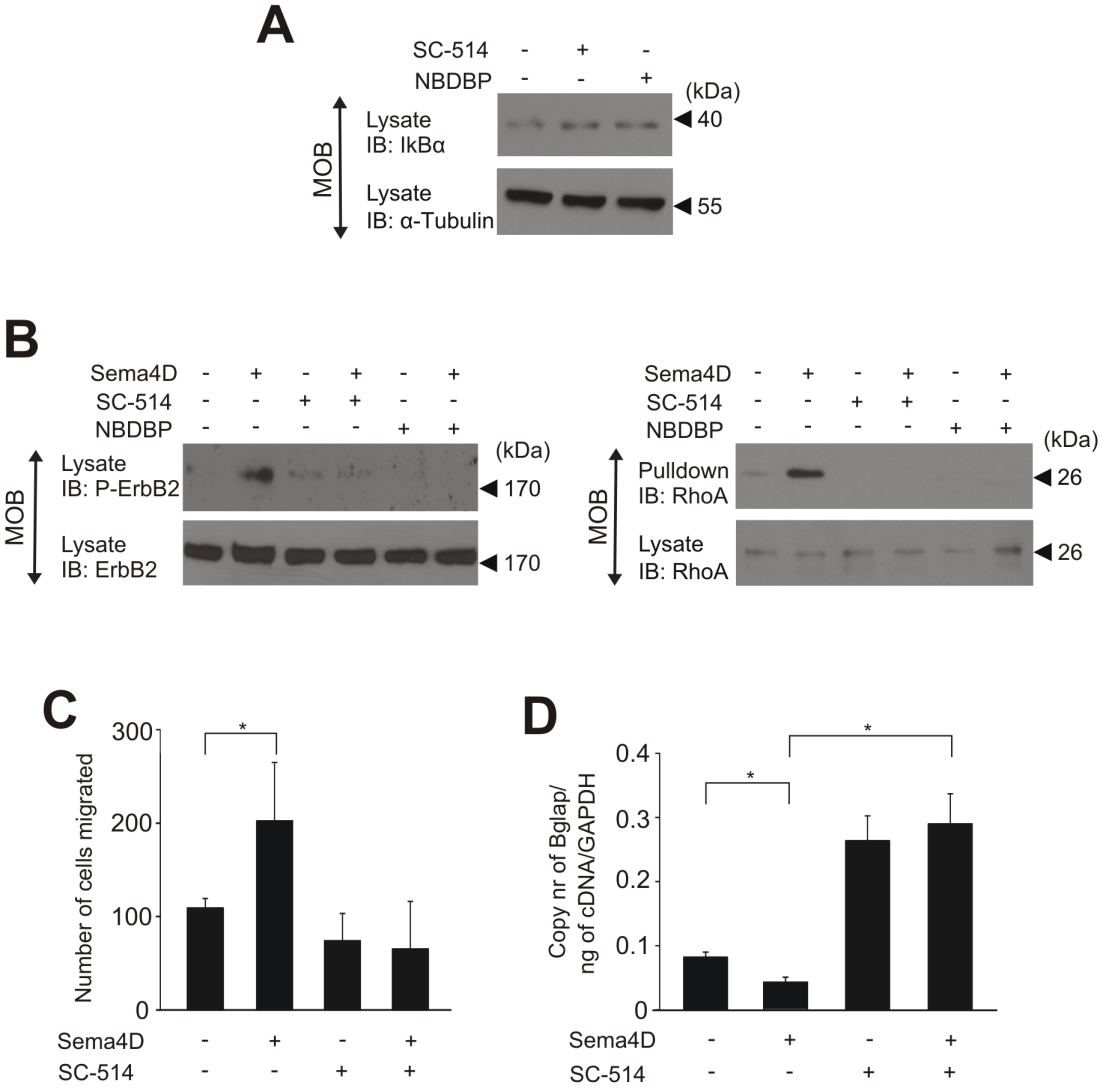
IKK-dependent Plexin-B1 signalling in mouse primary osteoblasts. (**A**) Mouse primary osteoblasts (MOB) were treated without or with SC-514 (25 µM) or NBDBP (13 µM) for 30 minutes, lysed and subjected to immunoblotting using anti-IκBα antibody. Equal loading of samples was tested using an anti-α-tubulin antibody. (**B**) Isolated MOBs were cultured in osteogenic medium for three days. After 12 hours of starvation, cells were treated without or with SC-514 or NBDBP as described above followed by incubation with buffer (−) or 20 nM of Fc-Sema4D for 20 minutes. Cells were lysed, and ErbB2 phosphorylation (left panel) and RhoA activation (right panel) were determined as described in the *Materials and Methods*. Shown are representative examples of at least three experiments. (**C**) MOB cells were starved in MEMα medium containing 0.5% FBS, detached, counted and seeded on the fibronectin-coated transwell migration plates. Cells were allowed to migrate in the absence (−) or presence (+) of 20 nM Sema4D and 25 µM SC-514 as indicated for 4 hours. Migrated cells were fixed in methanol, stained and counted. (**D**) Mouse primary osteoblasts were allowed to differentiate in osteogenic medium for 7 days. During the differentiation period, the cells were treated without or with SC-514 (25 µM) in the absence (−) or presence (+) of 150 nM of Sema4D. After 7 days, RNA was isolated from cells and the osteocalcin (Bglap) expression was determined by RT-PCR. Shown are mean values −/+ SD from triplicates. *, P<0.05.

The Sema4D-induced dedifferentiation of osteoblasts has been shown to be mediated by Plexin-B1-induced, ErbB-2-dependent RhoA activation [26]. In mouse osteoblasts we observed a basal IKK activity which was sensitive to inhibition of IKK-complex components (Figure 7A). Consistent with data obtained in tumour cells, Sema4D-induced ErbB-2 phosphorylation and RhoA activation in mouse osteoblasts were sensitive to the inhibition of the IKK-complex (Figure 7B). Finally, we found that Sema4D-induced migration of mouse osteoblasts and Sema4D-dependent osteoblast dedifferentiation were blocked by inhibition of IKKβ (Figure 7C and D), indicating that the IKK-complex controls plexin-B signalling also in osteoblasts.

## Discussion

Plexins are widely expressed transmembrane receptors that mediate the effects of semaphorins. In the past years some of the signalling mechanisms used by plexins have been described. Besides the regulation of R-Ras through a conserved GAP-domain, plexin-B family members mediate the activation of the small GTPase RhoA through their interaction with the guanine nucleotide exchange factors PDZ-RhoGEF and LARG. RhoA activation requires the association of B-plexins with the receptor tyrosine kinase ErbB-2. By performing a cellular siRNA-based assay to screen for protein kinases that are potentially involved in B-plexin-mediated RhoA-activation, we unexpectedly found that the IKK-complex is crucial for Sema4D-induced ErbB-2 phosphorylation and downstream signalling processes leading to the activation of the small GTPase RhoA. Activity of the IKK-complex is not only required for ErbB-2 phosphorylation, but also mediates the interaction of B-plexins with ErbB-2 under basal conditions. This function of the IKK-complex is specific for B-plexin-mediated RhoA activation, and the GAP-function of Plexin-B1 and Plexin-B2 was not affected by knockdown of IKK-subunits.

The IKK-complex plays a crucial role in the activation of the transcription factor nuclear factor kappa B (NF-κB) by phosphorylating the inhibitory molecule IκBα, which triggers its subsequent polyubiquitylation and degradation [44]. In recent years, evidence has been gathered indicating that IKKs do not only target upstream mediators in NF-κB cascades, but also proteins unrelated to NF-κB signaling, thereby mediating crosstalk with other signalling cascades [45]–[49]. We found that Sema4D-induced ErbB-2 phosphorylation and subsequent activation of RhoA was not affected by interfering with NF-κB directly, indicating that the regulation of signalling via B-plexins is a novel NF-κB-independent function of the IKK-complex. Whereas the IKK-complex is exclusively involved in canoncical NF-κB activation by phosphorylating IκBα, it has often been observed that NF-κB-independent effects of the IKK-complex are mediated by only one catalytic subunit, IKKα or IKKβ respectively [46],[50]. As B-plexin-mediated ErbB-2 phosphorylation and subsequent RhoA activation were completely inhibited by siRNA-mediated knockdown of each subunit of the IKK-complex, the complete IKK-complex is obviously required for signalling via B-plexin family members.

Catalytic IKK subunits regulate celluar processes by the phosphorylation of effector proteins containing the consensus sequence “DpSGψXpS/T” [34]. In addition, recent studies identified regulatory protein interactions of IKKα, which are independent of its kinase activity [51]–[54]. Overexpression of kinase-deficient IKKα and IKKβ mutants and incubation of cells with an IKKβ kinase inhibitor blocked B-plexin-mediated RhoA activation, thereby indicating that the serine kinase activity of both catalytic IKK-subunits are involved in signalling mechanisms mediated by B-plexins.

Our data indicate that serine phosphorylation by the IKK-complex is not only crucial for the trans-phosphorylation of ErbB-2, but also mediates the stable interaction of ErbB-2 with B-plexins under resting conditions. In HEK-293 cells transfected with Plexin-B1 and ErbB-2 mutants, which both lack the whole intracellular parts of the protein, the IKK-inhibitors SC-514 and NBDBP still decreased interaction of Plexin-B1 and ErbB-2, which is known to be mediated by their extracellular domains [22]. As the IKK-complex is present in the cytoplasm, a mechanism through which the IKK-complex induces direct serine phosphorylation of the extracellular parts of ErbB-2 or Plexin-B1 is hard to imagine. Our data therefore suggest that the association of ErbB-2 and Plexin-B1 requires another transmembrane protein that may serve as a substrate for the IKK-complex. However, this putative adaptor protein could not be identified using different approaches and therefore still remains unknown. Alternatively, lipid components or other non-protein components are required.

In contrast to endothelial cells, where Plexin-B1 was shown to activate NF-κB [55], we did not observe an activation of IKK or the NF-κB pathway in response to Sema4D in MCF-7 or HEK-293 cells. These results support the notion that downstream effectors of B-plexins depend on the cellular context and the expression of additional regulatory proteins. However, consistent with previous reports [43],[56], we detected an elevated kinase activity of catalytic IKK subunits in the investigated cell lines under basal conditions. Constitutively active IκB kinases have been described in various cancer cells including melanomas [41], prostate cancer [37],[39], pancreatic cancer [40], squamous cell head-neck-carcinoma [38] and mammary gland carcinoma [43]. The observation that IKKs are activated under basal conditions in different cell lines explains why B-plexin-mediated RhoA activation can be induced by semaphorins without additional IKK activation by other stimuli. However, we found that plexin B-mediated RhoA activation by semaphorin can be potentiated by additional IKK activation.

The fact that B-plexin-mediated ErbB-2 phosphorylation and subsequent RhoA activation depends on the IKK-complex and can be blocked by IKK inhibitors may suggest a potential therapeutic approach. ErbB-2-overexpressing tumours depend on Plexin-B1-mediated RhoA activation for tumour progression and metastasis [25]. In endothelial cells, activation of Plexin-B1 induces a pro-angiogenic response in a RhoA-dependent manner, which is of particular importance for the neovascularization of tumours [55],[57]. Recent evidence shows that Sema4D-induced Plexin-B1- and ErbB-2-dependent RhoA activation inhibits osteoblast differentiation resulting in reduced bone formation [26]. Interestingly, we observed that the Sema4D-dependent migration and dedifferentiation of osteoblasts requires IKK-complex activity, thereby indicating that the significance of the identified IKK signalling mechanism is not only restricted to cancer cells but also extends to other non-malignant cell types. Beside enhanced osteoclastic bone resorption, a decrease in osteoblastic bone formation is observed in bone loss associated with inflammatory and neoplastic diseases [58],[59]. Since Sema4D is expressed in T-cells and certain types of cancer cells, Plexin-B1-mediated and IKK-complex-dependent RhoA activation might contribute to reduced bone formation under these circumstances. The IKK-complex might therefore represent a novel therapeutic target for the treatment of B-plexin-dependent tumours as well as in osteoporosis and other bone diseases.

Recent studies have shown that the IKK-complex is critically involved in tumorigenesis and metastasis [47],[60]–[64]. Given that IKK activation enhances Plexin-B1-dependent activation of RhoA, which is known to subsequently increase the promigratory activity of cancer cells and to increase tumour progression [24],[25], the IKK-complex may exert some of its tumour-promoting activity through enhanced plexin signalling.

## Materials and Methods

### Antibodies and chemicals

The following antibodies were used and obtained from commercial sources: Mouse monoclonal anti-ErbB-2 (Invitrogen, 1∶1000), rabbit polyclonal anti-phospho-ErbB-2 (Y1248, 1∶400), rabbit monoclonal anti-RhoA (1∶400), rabbit polyclonal anti-R-Ras (1:400) and rabbit monoclonal anti-IκBα (Cell Signalling Technology, 1:400), mouse monoclonal anti-HA and mouse monoclonal anti-α-tubulin (Sigma-Aldrich, 1∶1000), goat monoclonal anti-Plexin-B1 (R&D Systems, 1∶400), rabbit polyclonal anti-IKKα/β (Santa Cruz Biotechnology, 1∶500), goat polyclonal anti-VSV-G (Thermo Scientific, 1∶1000), mouse monoclonal anti-Met (Invitrogen, 1∶1000). SC-514, SN50 and NBDBP were purchased from Calbiochem.

### Plasmids and viruses

The eukaryotic expression plasmid carrying the human cDNA of FLAG-PDZ-RhoGEF was described previously [24]. Human VSV-Plexin-B1 was kindly provided by L. Tamagnone (University of Torino, Turin, Italy). C-terminally truncated versions of human VSV-Plexin-B1 (VSV-Plexin-B1ΔIC) lacking amino acids 1514–2135 and human HA-ErbB-2 (HA-ErbB-2ΔIC) lacking amino acids 680–1255 were generated by PCR and cloned into pcDNA3. HA-IKKα-KD (K44M), HA-IKKβ-KD (K44M) and NF-κB-dependent luciferase reporter plasmid (NF-κB-Luc) were obtained from D. Brandt (University of Marburg, Marburg, Germany). The recombinant adenovirus expressing a dominant negative mutant of IκBα (IκBα-S32A/S36A), resistant to its phosphorylation-induced degradation, was obtained from Vector Biolabs. To generate the retroviral luciferase reporter plasmid RepLucdelCMV, the 3DA.Fos sequence encoding firefly luciferase under the control of a mutant serum response element (SRE.L), which lacks a ternary complex factor binding site [27], was amplified by PCR from 3DA.Luc, kindly provided by R. Treisman (London Research Institute, London, UK), and inserted into NotI and SalI sites in the ORF of the retroviral-based vector pLNCX2 (Clontech Laboratories). As the constitutively active CMV-promotor in pLNCX2 would have interfered with the inserted SRE.L, we amplified the backbone of pLNCX2 by PCR using two primers, which bind complementarily in the 3′ Late translated region and distal of the neomycin phosphotransferase cassette sparing the CMV-promotor, and religated the construct. The resulting retroviral reporter plasmid was confirmed by sequencing.

### Cell culture and transfection

HEK-293 and MDA-MB-468 cells were obtained from the American Type Culture Collection. MCF-7 and BT-474 cells were obtained from the German Collection of Microorganisms and Cell Cultures (DSMZ). The amphotropic cell line PT-67 was obtained from Clontech Laboratories. MT-2 cells were a kind gift from Michael Karin (University of California, San Diego [64]). HEK-293 cells were cultured as described previously [16]. MCF-7, MDA-MB-468, BT-474 and MT-2 cells were cultured as described before [23],[25],[65]. PT-67 cells were cultured according to the manufacturer's instructions (Clontech). HEK-293 cells were transfected with cDNA plasmids using the calcium phosphate method as described before (Swiercz, 2002). SiRNA transfections of BT-474 and MT-2 cells were carried out using Lipofectamine RNAiMAX (Invitrogen) according to the manufacturer's instructions. MCF-7 cells were transfected with siRNA using Hiperfect. For knockdown efficiency in MT-2 cells mRNA was isolated and the expression of Plexin-B1 was determined by quantitative RT-PCR.

Primary mouse osteoblast (MOB) cells were isolated from calvaria of 3-day-old female pups by sequential digestion with 0.2% collagenase and 0.2% dipase II. Cells from fraction 2 to 5 were pooled and grown in MEMα/10% FBS in 6-well plate. After 48 hours, cells were trypsinized, counted and seeded for the respective experiments. For Western blot and RhoA pulldown experiments, cells were cultured in osteogenic induction medium (MEMα, 10% FBS, 100 µg/ml ascorbic acid 5 mM β-glycerophosphate) for 3 days. After 12 hours of starvation in serum free medium, cells were treated with 25 µM SC-514 or 13 µM of NBDBP for 30 minutes and stimulated with control or 20 nM Fc-Sema4D (R&D Systems) for 20 minutes. The cells were then lysed in ice cold radioimmunoprecipitation (RIPA) buffer (1% Triton X-100, 150 nM NaCl, 50 mM Tris pH 7.4, 0.1% sodium dodecyl sulfate, 0.25% sodium deoxycholate, 1 µg/ml of each leupeptin, aprotinin and pepstatin, 1 mM 4-(2-aminoethyl)–benzosulfonylfluoridhydrochloride and 1 mM Na_3_VO_4_) for Western blot or lysed in 50 mM Tris, pH 7.2, 1% Triton X-100, 0.5% sodium deoxycholate, 0.1% SDS, 500 mM NaCl, 10 mM MgCl2, 1 µg/ml of each leupeptin, aprotinin and pepstatin, 1 mM 4-(2-aminoethyl)–benzosulfonylfluoridhydrochloride for RhoA pulldown [23], and the samples were processed for the respective methods.

For differentiation, MOB cells were cultured in osteogenic induction medium (MEMα, 10% FBS, 100 µg/ml ascorbic acid and 5 mM β-glycerophosphate) in 24 well plates under the treatment of 25 µM SC-514, 13 µM of NBDBP and Sema4D (150 nM) in the respective wells for 7 days. mRNA was isolated and the expression of osteocalcin (Bglap) was determined by quantitative RT-PCR.

Animals used for osteoblast isolation were sacrificed by cervical dislocation according to the guidelines approved by the local authorities (Regierungspräsidium Darmstadt, Hessen, Germany). This study was approved by the Animal Welfare Committee of the Regierungspräsidium Darmstadt, Hessen, Germany.

### Sema4D-signalling biochemistry

For immunoprecipitation, HEK-293 or MCF-7 cells were collected 48 hours after cDNA transfection or incubation with 50 µM SC-514 (Calbiochem) or 100 µM NBDBP (Calbiochem) for 30 minutes and lysed in ice-cold RIPA buffer (1% Triton X-100, 150 nM NaCl, 50 mM Tris pH 7.4, 0.1% sodium dodecyl sulfate, 0.25% sodium deoxycholate, 1 µg/ml of each leupeptin, aprotinin and pepstatin, 1 mM 4-(2-aminoethyl)-benzosulfonylfluoridhydrochloride and 1 mM Na_3_VO_4_). Proteins from cell extracts were immunoprecipitated using an anti-VSV-G-agarose affinity gel (Sigma-Aldrich). For precipitation of endogenous Plexin-B1 in MCF-7 cells, we used an anti-Plexin-B1 antibody (R&D Systems). Antibody-antigen complexes were isolated by binding to protein A/G-sepharose (Santa Cruz Biotechnologies) followed by washing with ice-cold radioimmunoprecipitation buffer.

Rho pulldown assays were performed 48 hours after siRNA/cDNA transfection. To determine ErbB-2 phosphorylation at tyrosine-1248, which was previously shown to indicate plexin-B activation [22], MCF-7 cells were either transfected with siRNAs directed against IKKα, IKKβ or IKKγ according to the screening protocol or incubated with IKK inhibitors SC-514 (50 µM) or NBDBP (100 µM) respectively, each for 30 minutes. Thereafter cells were stimulated without or with 150 nM Sema4D or incubated in the absence or presence of 10 ng/ml EGF (Cell Signalling Technology) and lysed in Laemmli buffer (62.5 mM Tris pH 6.8, 2% SDS, 10% glycerol, 0.001% brompenhole blue, 5% 2-mercaptoethanol). To study IκBα degradation, MCF-7 cells were incubated with TNFα (25 ng/ml) (Sigma-Aldrich) or Sema4D (150 nM) for increasing time periods and lysed in Laemmli buffer. Protein lysates or precipitated proteins were then separated using SDS-PAGE and transferred to nitrocellulose membranes. Non-specific binding sites were blocked with 5% milk in TBST. Blots were probed with the indicated antibodies, and proteins were visualized using enhanced chemiluminescence system (ECL) (GE Healthcare and Millipore).

### Retroviral infection and generation of MCF-7 reporter cell line

The reporter plasmid RepLucdelCMV was transfected into the packaging cell line PT67 (Clontech Laboratories) using the calcium phosphate method. Transfected PT67 cells were selected with 400 µg/ml Geneticin (Invitrogen) for 14 days. Viral supernatants were collected, filtrated through a 0.45-µm polyvinylidene difluoride filter (Millipore) and used to transduce 3×10^5^ MCF-7 cells in a 10-cm dish in the presence of 6 µg/ml polybrene (Sigma). The infected cells were selected with 400 µg/ml geneticin for 14 days. Hereafter, single MCF-7 reporter cell colonies were isolated, transferred into separate wells of a 96-well plate and analyzed for luciferase-responsiveness upon incubation with Sema4D (150 nM) or LPA (25 µM) for 8 hours using the ONE-Glo luciferase assay system (Promega) according to the manufacturer's instructions. Luciferase activity values were normalized to the number of viable cells using the CellTiter-Fluor cell viability assay (Promega). The MCF-7 reporter clone demonstrating the highest sensitivity was routinely used for all further screening experiments.

### siRNAs

Kinome-wide Silencer Select Human Kinase siRNA Library (V4) was purchased from Ambion. Control siRNA, showing no homology to any known mammalian gene and siRNAs targeting human IKKs (Cat nr. SI03650318, S100605115, S100605122, S100300545, S102777376, S102223333 and S102223340) were purchased from Qiagen. siRNA targeting human Plexin-B1 was described before [23].

### siRNA screen

The kinome-wide Silencer Select Human Kinase siRNA Library V4 (Ambion) was used for the screen. The library contained 2130 siRNAs targeting 710 protein kinases (3 siRNAs/kinase). Dissolved library was used to prepare replica plates containing 2 pmole of siRNA/well. As positive controls, functionally validated siRNAs directed against Plexin-B1 [23] and ErbB-2 (Qiagen) were spotted manually into two empty wells on each of the replica 96-well plates. Each plate also included one well containing transfection medium only, which served as blank, and two wells with a non-silencing siRNA (IBA GmbH, Germany) or an siRNA targeting the receptor tyrosine kinase Met (Qiagen), serving as negative controls. For reverse transfection, 1.5 µl HiPerFect transfection reagent (Qiagen) were diluted in 25 µl of serum-free medium, added to each well and incubated for 10 minutes at room temperature for liposomal complex-formation followed by the addition of 5×10^4^ MCF-7 reporter cells (diluted in 165 µl culture medium) for a final siRNA concentration of 10 nM. 48 hours after transfection, MCF-7 reporter cells were starved for 12 hours and incubated in the presence of 150 nM Sema4D for 8 hours. In a parallel experiment, MCF-7 reporter cells were stimulated with 25 µM LPA for the same time periode. Thereafter, cell viability was measured using CellTiter-Fluor (Promega), followed by a determination of luciferase activity using ONE-Glo (Promega). Thereafter, luciferase activity was normalized, and specific hits were defined as protein kinases, whose siRNA-mediated depletion resulted in a decrease of normalized SRE reporter luciferase activity >0.2 in only one pathway, Sema4D or LPA respectively, for at least 2 out of 3 siRNAs. Sema4D-specific gene targets were tested again using two independent siRNAs sequences from a different manufacturer (Qiagen). Confirmed hits were definied as those kinases whose siRNA-depletion caused a specific reduction of normalized SRE reporter luciferase activity >0.2 exclusively after Sema4D stimulation in at least 4 out of 5 independent siRNAs in total.

### Determination of activated RhoA and R-Ras

The amounts of activated cellular RhoA and R-Ras were determined by precipitation with a fusion-protein consisting of GST and the Rho-binding domain of Rhotekin (GST-RBD) or the Ras-binding domain of Raf1 (GST-Raf1) as described previously [66],[67]. All pulldown experiments were carried out 48 hours after siRNA transfection followed by overnight starvation in serum-depleted culture medium. Cells were incubated without or with 150 nM Sema4D or Sema4C for 20 minutes prior to cell lysis.

### In vitro kinase assay

MCF-7 cells were seeded onto 10-cm dishes and cultured in serum-depleted medium for 12 hours. After 20 minutes of incubation with control buffer (1× PBS), Sema4D (150 nM) or TNFα (25 ng/ml), cells were lysed in ice-cold radioimmunoprecipitation buffer (1% Triton X-100, 150 nM NaCl, 50 mM Tris pH 7.4, 0.1% sodium dodecyl sulfate, 0.25% sodium doxycholate, 1 µg/ml of each leupeptin, aprotinin and pepstatin, 1 mM 4-(2-aminoethyl)-benzosulfonylfluoridhydrochloride and 1 mM Na_3_VO_4_), and proteins from cell extractes were immunoprecipitated using an anti-IKKα antibody coupled to protein A/G-sepharose beads (Santa Cruz Biotechnology). The immunoprecipitates were washed four times in lysis buffer and subjected to an in vitro kinase assay using the HTScan IKKβ kinase assay kit (Cell Signalling Technology) according to the manufacturer's instructions. Briefly, 25 µl of each precipitated sample were preplated with 25 µl kinase reaction buffer (10 mM Tris-HCl pH 7.5, 2 mM beta-glycerophosphatem 0.8 mM dithiothreitol, 0.04 mM Na_3_VO_4_, 4 mM MgCl_2_), and phosphorylation was started by adding 25 µl of ATP (10 mM)/biotinylated IκBα-substrate solution. A recombinant, constitutively active IKKβ mutant (Cell Signalling Technology) served as positive control. After incubation at room temperature for 30 minutes, the reaction was stopped using 50 µl of 50 mM EDTA pH 8.0. For detection and quantification of phosphorylated IκBα-substrates, samples were transferred into separate wells of a streptavidin-coated 96-well plate and probed with an anti-phospho-IκBα (S32) antibody (Cell Signalling Technology). After three washing steps with 1x PBST and incubation with horseradish peroxidase-conjugated secondary antibody, 100 µl of TMB substrate (Cell Signalling Technology) were added and incubated at RT for 15 minutes. To stop the staining reaction 100 µl of Stop Solution (Cell Signalling Technology) were added per well and the serine-phosphorylation of IκBα was quantified using a Multiskan Spectrum Luminometer at a wavelength of 450 nm (Thermo Scientific).

### Migration and invasion assays

To measure cell migration, mouse osteoblasts (1×10^3^) in MEMα, 0.5% FBS were seeded into fibronectin (10 µg/ml)-coated 96 well migration chambers (Corning). SC-514 (25 µM) or NBDBP (13 µM) alone or together with Sema4D (150 nM) was added to the lower chamber of the respective wells. The cells were then allowed to migrate for 4 hours and the migrated cells at the lower surface of the filter were fixed in methanol, stained using toluidine blue and counted. For the determination of cell invasiveness, overnight-starved BT-474 (1×10^5^) or MT-2 (3×10^4^) cells were placed in transwell invasion inserts (Corning). BT-474 cells were treated with SC-514 (25 µM) or NBDBP (13 µM). After 24 hours, cells at the lower surface of the invasion filter were fixed, stained with Hoechts 33342 (DAKO) and counted.

### Statistical analysis

Quantitative data are given as mean values ± SD from, at least, three independent experiments. The statistical significance was evaluated by Student's t-test. Significance levels are indicated in the figure legends.
